# Vitamin D and adrenal gland: Myth or reality? A systematic review

**DOI:** 10.3389/fendo.2022.1001065

**Published:** 2022-10-13

**Authors:** Antonella Al Refaie, Leonardo Baldassini, Michela De Vita, Stefano Gonnelli, Carla Caffarelli

**Affiliations:** Division of Medicine, Department of Medicine, Surgery and Neuroscience, University of Siena, Siena, Italy

**Keywords:** vitamin D, VDR polymorphisms, adrenal gland, Cushing’s syndrome, adrenal tumor, hyperaldosteronism, primary adrenal insufficiency

## Abstract

In recent years, vitamin D has become the protagonist in many studies. From cardiology to oncology the spotlight was on this vitamin. While in the past it was considered for its important role in phospho-calcium metabolism and skeletal disorders; today by studying it better, thousands of scenarios and facets have opened up on this vitamin which is actually a hormone in all respects. There are authoritative studies that demonstrate its activity *in vitro* and *in vivo* on: carcinogenesis, inflammation, autoimmunity and endocrinopathies. Its role has been studied in type 1 and type 2 diabetes mellitus, in Hashimoto or Graves’ thyroiditis and even in adrenal gland diseases. In fact, there are several studies that demonstrate the possible correlations between vitamin D and: Addison’s disease, Cushing disease, hyperaldosteronism or adrenocortical tumors. Moreover, this fascinating hormone and adrenal gland even seem to be deeply connected by common genetic pathways. This review aimed to analyze the works that have tried to study the possible influence of vitamin D on adrenal diseases. In this review we analyze the works that have tried to study the possible influence of vita-min D on adrenal disease.

## Introduction

Vitamin D has a fundamental role in mineral and bone metabolism: it promotes calcium absorption in the gut and maintains adequate serum calcium and phosphate concentrations to enable normal bone mineralization. It is also needed for bone growth and bone remodeling.

**Figure 1:**
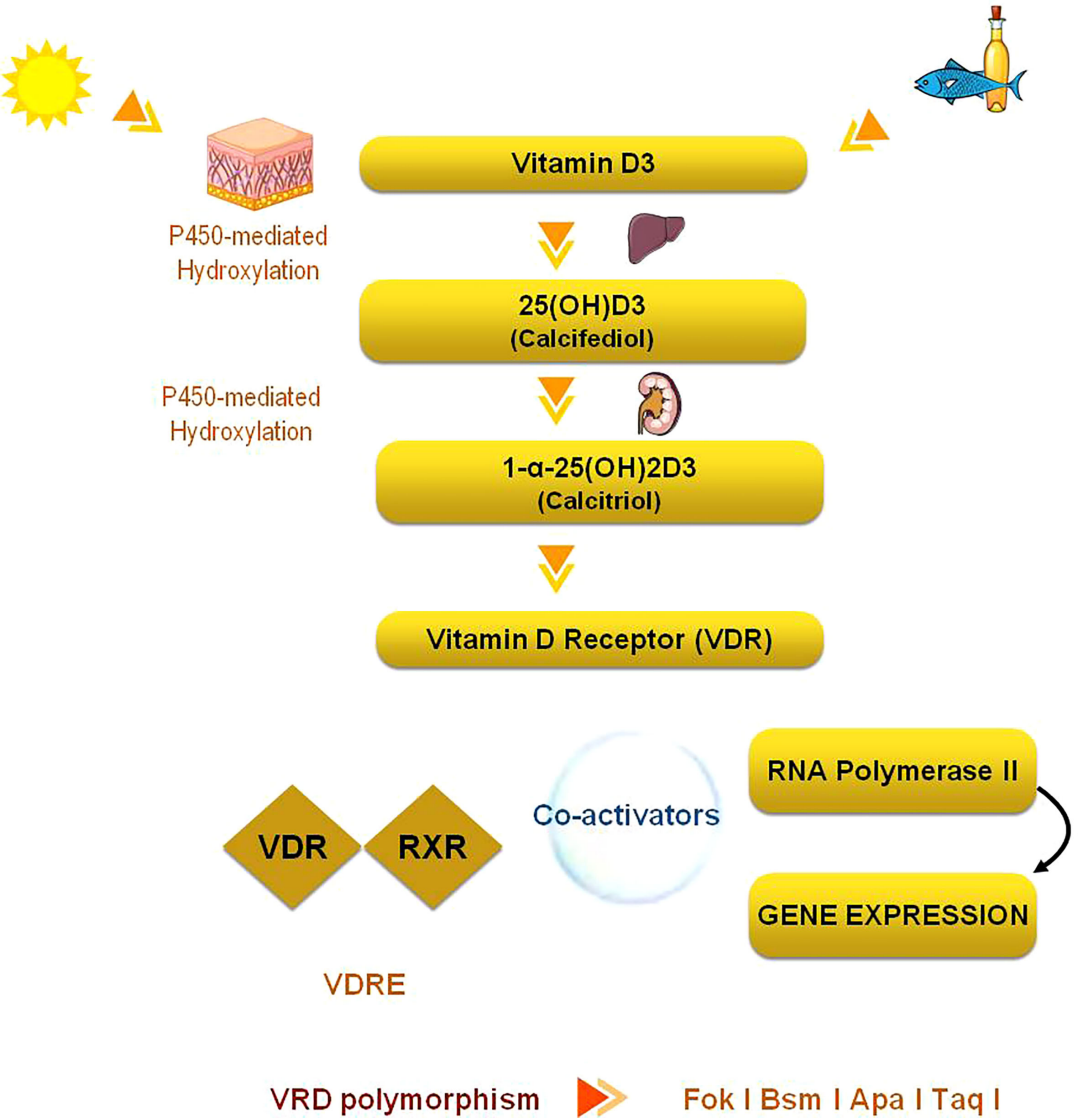
The pathways of production and regulation of Vitamin D.

The production of vitamin D3 (cholecalciferol) in the skin is not an enzymatic process: cholecalciferol is a steroid hormone precursor synthesized in skin thanks to a photochemical reaction by ultraviolet B radiation of sunlight. Cholecalciferol is biologically inactive and needs two successive hydroxylation reactions for activation. The first hydroxylation occurs in the liver to form the 25-hydroxyvitamin D [25(OH)D or calcidiol]; the second occurs in the kidney to form 1,25-dihydroxyvitamin D, the biologically active vitamin, [1,25(OH)2D or calcitriol] this hydroxylation is promoted by the 1α-hydroxylase (CYP27B1), an enzyme which is dependent on parathyroid hormone (PTH). 25OHD and 1,25(OH)2D catabolism depends on CYP24A1 (1, 2) **Figure 1**.

Parathyroid hormone (PTH), calcium, phosphate and Fibroblast Growth Factor-23 (FGF-23) are the major regulators of the renal 1-hydroxylase (CYP27B1); regulation of the extra renal 1-hydroxylase is different from the kidney one and implies cytokines. Vitamin D and its metabolites are carried in the blood by albumin and vitamin D binding protein (DBP). Most, but not all actions of 1,25(OH)2D are mediated by the vitamin D receptor (VDR). VDR is a transcription factor that interacts with other transcription factors; calcitriol binds the nuclear VDR and regulates positively or negatively the transcription of several target genes depending on other cofactors to which it binds or relates. The VDR is found in most cells, not just those involved with bone and mineral homeostasis suggesting many actions of 1,25(OH)2D on different physiologic and pathologic processes (3, 4). The expression of 1α-hydroxylase and VDR in tissues involved in classical endocrine pathway, in breast, pancreas, colon, in malignant cells and immune cells, suggests that also other tissues, different from kidney, are able to synthesize the active form of vitamin D (5). So vitamin D has pleiotropic effects and may act in paracrine and autocrine manner in addition to its endocrine function. This suggests an important role of vitamin D in the pathogenesis and outcome of many common diseases, including endocrine diseases (6), chronic diseases (7) and cancer (8).

This review aimed to analyze the works that have tried to study the possible influence of vitamin D on adrenal diseases.

## Materials and methods

We searched the MEDLINE/PUBMED, Cochrane Library, ClinicalTrials.gov and SCOPUS databases up to 1975 to 2022 to identify all relevant English language medical literature for studies under the search text terms; Vitamin D AND (adrenal gland OR Cushing’s syndrome OR adrenal tumors OR primary aldosteronism OR primary adrenal insufficiency).

Across these databases, these search terms produced 942 results.

**Figure 2:**
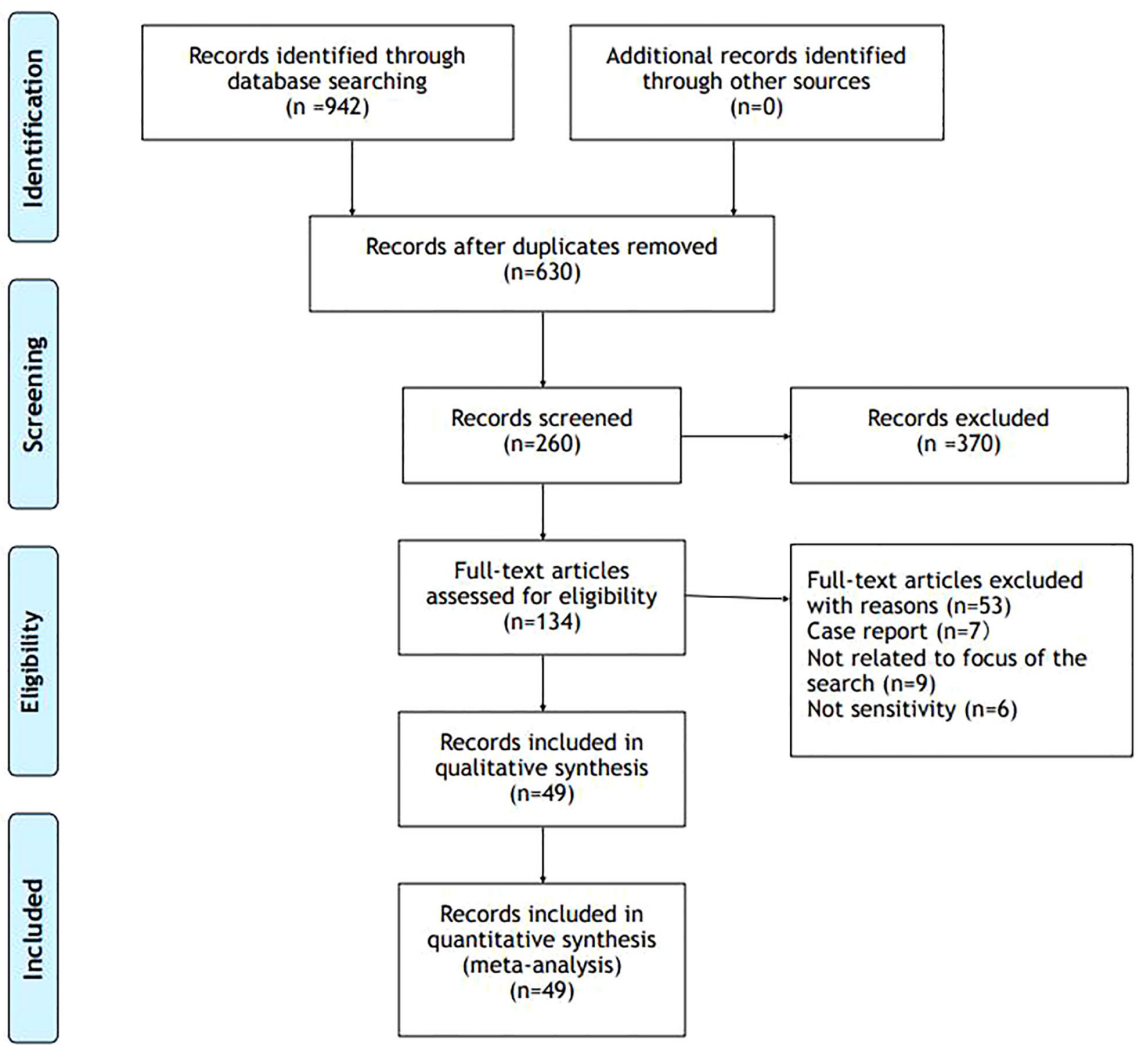
PRISMA Flow Diagram.

From these results, we excluded studies about adrenal medulla (74) and we excluded duplicates. We did a screen considering predominantly works that matched adrenal gland cortex and vitamin D (260). Among these works we select articles with greater rigor, we excluded case reports and articles not focus on the topic **Figure 2**.

## Vitamin D and Cushing’s syndrome

Cushing’s syndrome (CS) is a rare endocrine disease characterized by high circulating levels of cortisol. There are two etiologies of CS: endogenous and exogenous. The most common cause of exogenous hypercortisolism is iatrogenic and depends on the prolonged use of glucocorticoids (GCs). Endogenous hypercortisolism depends on excessive production of cortisol by adrenal glands and it can be ACTH-dependent or ACTH-independent. ACTH-secreting pituitary adenomas (Cushing disease) and ectopic ACTH secretion by neoplasms are responsible for ACTH-dependent Cushing. Adrenal hyperplasia, adenoma and carcinoma are major causes of ACTH-independent Cushing syndrome. CS is causes severe morbidities and increases mortality, the most common complications are the cardiovascular ones. Signs and symptoms of CS are: severe fatigue, depression, anxiety, irritability, muscle weakness, cognitive difficulties, new or worsened high blood pressure, infections, skin darkening, bone loss, fractures, decreased sex drive, decreased fertility, erectile dysfunction, hirsutism, weight gain, “moon face”, “buffalo hump”, pink or purple stretch marks (striae) on the skin of the abdomen, thighs, breasts and arms, thinning, fragile skin that bruises easily, slow healing of cuts, infections, acne.

The relationship between CS and vitamin D is certainly the most complex and ambiguous one. The relationship between vitamin D and GCs has been extensively studied and the possibility that the use of GCs would alter the bioavailability of vitamin D has also been investigated (9, 10). The mechanism by which the GCs reduce the levels of 25 (OH) D seems indirect in fact GCs regulate the expression of the vitamin D receptor in many tissues and cells (11, 12). Studies in mice showed that use of dexamethasone was associated with a decrease in 1α-hydroxylase and an increase in 24-hydroxylase, which can lower the active form of calcitriol, thus decreasing the circulating levels of 25 (OH)D (13, 14).

The most important studies evaluating the Vitamin D and Cushing syndrome are summarized in **Table 1**.

**Table 1:** Main characteristics of the 4 studies included in the review on Cushing Syndrome and Vitamin D.

Study/years	Study Characteristics	Main results	Conclusion
Seeman E (1980) (15)	6 ♀ (age 42 yrs) with endogenous CS 6 ♀ (age 62 yrs) and 2 ♂ (age 51 yrs) with exogenous GCs for connective tissue disorders	excess of GCs causes small decreases in plasma concentrations of 25(OH)D but no significant changes in plasma 1,25(OH)D and PTH	GCs excess does not affect plasma levels, production, or degradation of 1,25(OH)2D in humans.
Kugai N (1986) (14)	7 ♀ (OP+) (42.7 ± 8.3 yrs) 7 ♀ and 3 ♂ (OP-) (33.8 ± 8.9 yrs)	In CS patients, plasma levels of 25OH vitamin D and 24.25 (OH) vitamin D were lower than in normal subjects	These findings seem to favor the mechanism of impaired calcium absorption involving abnormal vitamin D metabolism rather than the direct effects of GCs excess
Povaliaeva A (2021) (16)	CD: 26 ♀ - 4 ♂ (39.1 yrs) Controls: 26 ♀ - 4 ♂ (33.4 yrs) All participants receive a single dose (150,000 IU) of oral cholecalciferol.	CD 25(OH)D = 13.1 ng/mL Controls 25(OH)D = 21.7 ng/mL	Lower levels of free 25(OH)D observed in CD patients at baseline After treatment with cholecalciferol in patients with CD patients have a consistently higher 25 (OH) D/24.25 (OH) 2D ratio, which is indicative of a decrease in 24-hydroxylase activity. It concludes that this altered activity of vitamin D catabolism can influence the effectiveness of treatment with cholecalciferol
Guarnotta V (2022) (17)	CD: 43 ♀ - 7♂ (50.9 ± 17.4 yrs) Controls: 33 ♀ - 7♂ (48.5 ± 13.4 yrs) All participants receive a single dose (150,000 IU) of oral cholecalciferol.	CD 25(OH)D=16.7 ± 8.18 ng/mL Controls 25(OH)D= 28.7 ± 8.49 ng/mL	25(OH)D serum levels are lower in subjects with active CD compared to controls matched for age, BMI and gender. Vitamin D deficiency is correlated with mUFC and values of mUFC > 240 nmol/24 h are predictive of 25(OH)D deficiency. Cholecalciferol supplementation has a positive impact on insulin sensitivity and lipids.

♀: female; ♂: male; CS: Cushing’s syndrome; GCs: glucocorticoids; 25(OH)D: 25-hydroxyvitamin D; 1,25(OH)D: 1,25-dihydroxyvitamin D; PTH: parathyroid hormone; OP: osteoporosis; CD: Cushing’s disease; BMI: Body Mass Index; mUFC: mean urinary free cortisol.

Between 70’s-80’s there was strong interest in glucocorticoids and vitamin D. Some works reported during GCs therapy, values ​​of 1,25(OH)2D reduced, others increased, others unchanged (15, 18–20). The study by Seeman et al. in 1980 considered 14 patients with excess of endogenous or exogenous glucocorticoids. The dosage of vitamin D was performed both during euglucocorticoidism and during hyperglucocorticoidism; excess glucocorticoids was observed to cause small decreases in plasma concentrations of 25(OH)vitamin D (22 +/- 2-18 +/- 2 ng/ml; P <0.05) but no significant change in 1, 25-OHvitamin D (32 +/- 8- 23 +/- 6 pg/ml) (15). Another study by Kugai et al. (14) analyzed the impaired mineral metabolism and vitamin D metabolism in CS patients. In CS patients, plasma levels of 25OH vitamin D and 24,25(OH) vitamin D were lower than in normal subjects.

A recent study by Povalieva et al. evaluated vitamin D metabolism in patients with Cushing disease (CD) compared to healthy subjects receiving cholecalciferol bolus treatment. It studied: 30 adults with CD and 30 apparently healthy adults with similar age, gender and BMI. All participants received a single dose (150,000 IU) of oral cholecalciferol. The following data were determined: the serum metabolites of vitamin D, free 25 (OH) D, vitamin D binding protein (DBP) and PTH serum and urine biochemical parameters were performed prior to intake and on days 1, 3 and 7. The CD patients had lower baseline free 25 (OH) D levels (p <0.05) despite similar DBP levels (p>0.05) and lower albumin levels (p<0.05); 24-hour urinary free cortisol showed a significant correlation with the baseline 25 (OH) D/24.25 (OH) 2D ratio (r = 0.36, p <0.05). CD patients had a consistently higher 25(OH)D/24.25(OH) 2D ratio, which is indicative of a decrease in 24-hydroxylase activity. It was concluded that this altered activity of vitamin D catabolism could have influenced the effectiveness of treatment with cholecalciferol (16).

An even more recent study by Gurnotta et al. conducted on patients with CD is of considerable interest (17). The objectives of the study were to: **1**. evaluate serum 25-OH- vitamin D in CD patients, compared to controls; **2**. evaluate the response to a dose of 150,000 UI of cholecalciferol in patients with CD. We considered 50 patients with CD and 48 controls, in which the following data were evaluated: the anthropometric and biochemical parameters at baseline and after 6 weeks of therapy with cholecalciferol. At baseline, CD patients had a higher frequency of hypovitaminosis D deficiency (p=0.001) and lower serum 25(OH)D (p <0.001) than controls. Six weeks after cholecalciferol treatment, CD patients had increased serum calcium (p=0.017), 25 (OH) D (p<0.001), ISI-Matsuda (p=0.035), oral disposition index (p=0.045) and decrease in serum PTH (p = 0.004) and total cholesterol values ​​(p=0.017) from baseline. Mean urinary free cortisol (mUFC) was negatively correlated independently with 25 (OH) D in CD. In conclusion, serum levels of 25 (OH) D were lower in patients with CD than in controls. Vitamin D deficiency is correlated with mUFC and mUFC values>240 nmol/24 h are associated with hypovitaminosis D. The supplementation of cholecalciferol had a positive impact on insulin sensitivity and lipids (17).

From this analysis, we can conclude that the data on CS and vitamin D are ambiguous and not unique. Some of these studies have been conducted on humans, others on animal models, but only a few studies have been conducted on subjects with endogenous hypercortisolism such as the study of Guarnotta et al. probably also this makes the reading of the data more ambiguous; the results obtained in Guarnotta’s study are very promising and open a window on future works and possible discoveries.

## Vitamin D and adrenal tumors

The term “adrenal tumor” describes benign and malignant mass lesions of the adrenal gland. The estimated incidence of adult adrenocortical carcinoma (ACC) is between 0.7 and 2.0 per million per year. ACC can occur at any age with a peak incidence between 40 and 60 years, women are more often affected (55-60%). ACC can be sporadic or part of familial cancer syndrome (Li-Fraumeni syndrome, Lynch syndrome, multiple endocrine neoplasia (MEN) 1 and familial adenomatous polyposis). Most of ACCs are sporadic. Although the molecular mechanisms of tumorigenesis in many of the hereditary syndromes are well known, the molecular pathogenesis of sporadic ACCs is not completely understood. ACC can present classically in three different ways: about one-third of patients present with symptoms of hormonal excess (hypercortisolism, hyperandrogenism or both), another third present with non-specific symptoms and the last third are diagnosed coincidentally when imaging studies are done for other medical conditions (21, 22).

In recent years there has been a lot of attention on the possible role of Vitamin D in oncology (23). Laboratory and animal studies have reported that vitamin D can inhibit carcinogenesis, slow tumor progression, including promotion of cell differentiation, inhibition of tumor cell proliferation; moreover, vitamin D would also seem to have anti-inflammatory, immunomodulatory, proapoptotic and antiangiogenic effects (24). The literature data are many and the results are really promising. Numerous studies have confirmed a significant benefit of vitamin D in reducing mortality (25, 26). In particular, several studies, reviews and meta-analyzes have shown a positive effect of vitamin D on breast (27), colon (28–30) and prostate cancer.

In the **Table 2** we reported the most important studies about the possible effects of vitamin D on tumors of the adrenal gland.

**Table 2:** Main characteristics of the 4 studies included in the review on Adrenal Tumors and Vitamin D.

Study	Study characteristics	Main Results	Conclusions
Pilon C (2014) (31)	human H295R ACC cell line as model for analysis the role of 1,25(OH)D-VDR axis in the growth of ACC	first confirmed the presence of VDR in H295R cells by mRNA expression, western blot and immunofluorescence, with VDR displaying a diffuse nuclear localization Slightly supra-physiological concentrations of 1,25(OH)2D3 have a moderate antiproliferative effect on H295R cells	Protective role of VDR in the carcinogenesis of tumors of the adrenal cortex. VDR mRNA are lower in ACC
Pilon C (2015) (32)	ACC tissue samples of 12♀ - 11♂	Methylation in the promoter region of VDR gene was found in 3/8 ACCs, while no VDR gene methylation was observed in normal adrenals and adrenocortical adenomas. VDR mRNA and protein levels were lower in ACCs than in benign tumors, and VDR immunostaining was weak or negative in ACCs.	The association between VDR gene promoter methylation and reduced VDR gene expression is not a rare event in ACC, suggesting that VDR epigenetic inactivation may have a role in adrenocortical carcinogenesis.
Rubin B (2020) (33)	*in vitro* model with H295R ACC cells were tested with multiple concentrations of mithotane and 1,25 (OH)D for 24-96h and the results were analysed by MTT 2020	A reduction in cell growth was observed for both mitotane and 1α,25(OH)2D3. A combination of clinically sub-therapeutic concentrations of mitotane with 1α,25(OH)2D3, had an additive anti-proliferative effect	An additive anti-proliferative effect of vitamin D on inhibition growth of H295R ACC cells. A possible presence of a functional link between the VDR and Wnt/beta-catenin pathways
Bueno A C (2022) (34)	108 pediatric adrenocortical tumors 76 controls Evaluation of VDR mRNA (qPCR) and protein expression and VDR-wide methylation	Tumors with high VDR methylation presented lower mRNA levels and the respective patients presented advanced disease and reduced disease-free and overall survival	A role of VDR in normal adrenocortical development and homeostasis, which is impaired during tumorigenesis. Hypermethylation and under-expression of VDR can be both predictive and prognostic biomarkers for pACT

25(OH)D: 25-hydroxyvitamin D; 1,25(OH)D: 1,25-dihydroxyvitamin D; VDR: vitamin D receptor; ♀: female; ♂: male; MTT: 3-(4,5-dimethylthiazol-2-yl)-2,5-diphenyltetrazolium bromide; 1α,25(OH)2D3: 1α,25-dihydroxyvitamin D3; pACT: pediatric adrenocortical tumors.

One of the fundamental studies on this topic was that by Pilon et al. (31). This study analyzed the role of the Vitamin D-VDR receptor axis in the growth of ACC, using the H295R human adrenocortical carcinoma cell line as a model. It was concluded that: slightly increased concentrations relative to physiological standards of 1,25(OH)2D resulted in a moderate anti-proliferative effect on H295R cells. The anti-proliferative effect was due to the arrest of the cell cycle in the G1 phase, without inducing apoptosis. VDR mRNA expression was lower in ACC than in benign adrenocortical lesions and VDR immunostaining was evident in benign lesions while it appeared weak in tumor tissues. These findings may suggest a protective role of VDR in the carcinogenesis of tumors of the adrenal cortex (31).

Another recent study by Rubin et al. aimed to evaluate the effects of mitotane (the only chemotherapeutic agent available for the treatment of ACC) and 1,25(OH)2D, singly or in combination, in an *in vitro* model with ACC H295R cells, and to elucidate the molecular events behind their effects involving the Wnt/beta-signaling of the chain. Multiple concentrations of mitotane and 1,25(OH)2D, were tested on H295R cells for 24–96 hours and the effects were analyzed by 3-(4,5-dimethylthiazol-2-yl)-2,5-diphenyltetrazolium bromide (MTT). A reduction in cell growth in a dose/time dependent manner was observed for both mitotane and 1,25(OH)2D. Furthermore, a combination of clinically subtherapeutic concentrations of mitotane with 1,25(OH)2D, had an additive anti-proliferative effect, thus showing an additive effect of mitotane and 1,25(OH)2D on inhibition growth of H295R ACC cells and suggested the presence of a functional link between the VDR and Wnt/beta-catenin pathways (33).

A paper by Pilon et al. aimed to analyze the methylation of GpG sites in the promoter of the VDR gene of a different and wider range of human adrenocortical tissues, comparing adrenocortical adenomas (ACA) with ACC tissues. The study considered: 3 normal adrenal and 23 adrenal cortical tumor samples (15 adenomas and 8 carcinomas). While recognizing the limitations of the study (low number of the sample analyzed) the results obtained are very interesting, in fact it is the first evidence of an association between the methylation of the promoter of the VDR gene and the reduced expression of VDR in the ACC. This suggests a potential role in the epigenetic inactivation of VDR in malignant adrenocortical tumorigenesis. The authors also set VDR promoter methylation as a possible target for pharmacological agents for the treatment of adrenal cancer in selected cases (32).

Starting from the conclusions of this paper, a very recent retrospective cross-sectional study by Bueno et al. enrolling pediatric patients with pediatric adrenocortical tumors (pACT) and studied VDR expression levels and methylation status in pACTs and their clinical and prognostic significance. It considered clinical and pathological features, mRNA (qPCR) and protein (immunohistochemistry) expression of VDR and methylation at the VDR level of adrenocortical tumor samples from 108 pediatric patients. Fourteen pediatric and 32 normal fetal and postnatal adrenal glands were used as controls. Tumors with high VDR methylation had lower mRNA levels and their patients had advanced disease and reduced disease-free survival. The study therefore concluded that VDR has a role in normal adrenocortical development and homeostasis, which is impaired during tumorigenesis. Hypermethylation and under-expression of VDR can be both predictive and prognostic biomarkers for pACT (34).

Studies in literature that investigate the correlation between vitamin D and tumors of the adrenal cortex, although few, seem to have a single direction, underlining the important role of VDR in carcinogenesis. The results are very promising; they should lay the basis for future clinical studies. It would be interesting to be able to investigate the effect of vitamin D reintegration in the prevention of these tumors or in the outcome in patients with ACC through longitudinal clinical studies.

## Vitamin D and primary aldosteronism

The primary aldosteronism (PA) is the most common endocrine secondary form of hypertension, it represents the cause of the ≈5%-10% of hypertension in adults. Hyperaldosteronism can initially present as essential and refractory hypertension; it often goes undiagnosed and recent studies indicate that its prevalence may be at least 3-fold higher (35, 36). PA consists in a heterogeneous group of familial and sporadic disorders characterized by hypertension secondary to overproduction of aldosterone, low plasma renin activity, and low potassium levels. Symptoms can be: weakness, muscle spasms, temporary paralysis, tingling feelings and frequent urination. Sporadic PA is the most common form and it is caused by: Aldosterone Producing Adenoma (APA), unilateral adrenal hyperplasia (UAH) and bilateral adrenal hyperplasia (BAH) (37). PA is also linked to cardiovascular diseases and metabolic alterations (38). In literature, there are several *in vitro*-and-*in vivo* studies that suggest a connection between vitamin D and hyperaldosteronism.

The most important studies evaluating the Vitamin D and primary aldosteronism are summarized in **Table 3**.

**Table 3:** Main characteristics of the 13 studies included in the review on Hyperaldosteronism.

Study	Study Characteristics	Main Results	Conclusions
Yan Chun Li (2002) (39)	Mice VDR–/– Mice Gcm2–/–	Mice VDR–/– have major expression of renin and angiotensin II. In wild-type mice, the inhibition of 1,25(OH)2D leads to an increase in the renin expression, while the injection of 1,25(OH)2D leads to renin suppression	Vitamin D functions as a novel negative endocrine regulator of the renin-angiotensin system in animals. Maintaining a normal level of serum 1,25(OH)2D3 is important not only for calcium homeostasis, but also for the homeostasis of electrolytes, volume, and blood pressure
Yuan W (2007) (40)	mouse Ren-1c gene promoter by luciferase reporter assays	1,25(OH)2D3 suppresses renin gene expression at least in part by blocking the formation of CRE-CREB-CBP complex	1,25(OH)2D down-regulates renin gene transcription by suppressing, at least in part, CRE-mediated transcriptional activity in the renin gene promoter
Forman JP (2010) (41)	96 ♀ - 88♂ (40.1 ± 12.0 yrs)	25(OH)D = 22.1 ng/mL individuals with insufficiency and deficiency 25OHD had higher circulating angiotensin II levels	Low plasma 25(OH)2D levels upregulate RAS system. These finding may partly explain the higher risk of hypertension in individuals with vitamin D insufficiency or deficiency
Lundqvist J (2010) (42)	Human adrenocortical NCI-H295R cells treated for 24 h with 1α,25OH vitamin D3	The 1α,25-OHD3-mediated decrease in corticosterone and androgen production is due to suppression of the 21-hydroxylase activity by CYP21A2 and the 17,20-lyase activity by CYP17A1, respectively.	1,25(OH)D affects the production of hormones and expression of crucial steroidogenic enzymes in the human adrenocortical cell line NCI-H295R
Pilz S (2012) (43)	EH: 108 ♀ - 74♂ (50.2 ± 15.7 yrs) PA: 6 ♀ - 4♂ (50.1 ± 11.0 yrs)	EH: 25(OH)D=30.5 ± 15.0 ng/ml EH: PTH =46.5 ± 20.9 pg/ml PA: 25(OH)D=30.5 ± 15.0 ng/ml PA: PTH = 67.8 ± 26.9 pg/ml	PTH levels were significantly higher in PA patients compared with EH. Serum 25-OHD concentrations were similar in both group. PA patients are prone to secondary hyperparathyroidism that can be suc cessfully treated with MR antagonists or adrenal surgery
Maniero C (2012) (44)	APA: 14 ♀ - 33♂ (50 ± 13 yrs) EH: 29 ♀ - 32♂ (50 ± 15 yrs)	APA: 25(OH)D=34 ± 21 nmol/L APA: PTH = 118 ± 13 ng/L	25-OH-vitamin D levels in both groups are equally insufficient
Salcuni AS (2012) (45)	PA: 7♀ - 4♂ (56.0 ± 9.3 yrs) nPA: 10 ♀ - 5 ♂ (56.7 ± 9.5 yrs)	PA: 25(OH)D=32.2 ± 13.0 nmol/L PA: PTH = 9.8 pmol/L nPA: 25(OH)D=35.0 ± 17.8 nmol/L nPA: PTH = 5.3 pmol/L	Vitamin D levels are deficient in both groups, in PA group they are lower but not statistically significant. PA patients had a significantly higher PTH. PA is associated with low bone mass, increased prevalence of osteoporosis and vertebral fractures. Aldosterone excess lead to bone damage
Bi C (2013) (46)	microarray expression profiles of normal and APA samples from a public functional genomics data repository Gene Expression Omnibus (GEO, http:// www.ncbi.nlm.nih.gov/geo/) database	the VDR is the most significant transcription factor screened from the TF-target regulatory network and its target genes including CYP11B2 and KCNJ5	VDR is the most significant transcription factor and it might play a role in the endocrine mechanisms of APA
Ceccoli L (2013) (47)	EH: 75♀ - 35♂ (55.0 ± 10.0 yrs) PA: 51♀ - 66♂ (51.6 ± 11.0 yrs)	EH: 25(OH)D=26 ± 18 ng/ml EH: PTH =56.4 ± 16.4 pg/ml PA: 25(OH)D=24 ± 15 ng/ml PA: PTH = 82.2± 33 pg/ml In PA patients, PTH levels significantly increased with comparable vitamin D levels.	In PA after medical or surgical treatment PTH levels are significantly reduced and BMD significantly increased. 25 OH vitamin D levels are unchanged before and after the treatment. There isn’t a statistically significant difference between 25-OH vitamin D levels in both group
Petramala L (2014) (48)	EH: 38♀ - 35♂ (55.6 ± 12.4 yrs) PA: 22♀ - 51♂ (52.5 ± 11.2 yrs) Controls: 24♀ - 16♂ (55.7 ± 6.1yrs)	EH: 25(OH)D=32.9 ± 12.8 ng/ml EH: PTH =30.7 ± 11.9 pg/ml PA: 25(OH)D=17.8 ± 12.5ng/ml PA: PTH = 48.9 ± 19.9 pg/ml Controls: 25(OH)D=23.8 ± 12.8ng/ml Controls: PTH = 29.1± 2.4 pg/ml	Moreover, PA patients showed higher plasma PTH, lower serum 25(OH)-vitamin D levels, higher prevalence of vitamin D deficiency, and higher prevalence of osteopenia/osteoporosis than EH and controls. These observations support the hypothesis that bone loss and potentially fracture risk in PA patients are potentially the result of aldosterone mediated hypercalciuria and the consecutive secondary hyperparathyroidism
Zhang LX (2016) (49)	APA: 40♀ - 44♂ (50 ± 10 yrs) NFA: 37♀ - 21♂ (55 ± 8 yrs)	APA: 25(OH)D=11.5 ± 6.1 ng/ml APA: PTH =77.6 ± 34.8 pg/ml NFA: 25(OH)D=13.3 ± 6.9 ng/ml	Vitamin D levels are low in the all the 2 groups, in patients with APA levels are slightly lower but not statistically significant. PTH levels were significantly increased in patients with APA. The baseline and positional variation of PTH levels are significant in APA, thus PTH may be a promising auxiliary index for the clinical diagnosis of APA.
Grübler MR (2016) (50) longitudinal	EH Vit D: 45♀ - 47♂ (60.5 ± 10.9 yrs) EH Placebo:49♀- 46♂(59.7 ± 11.4yrs) 2800 IU of vitamin D3 for 8 weeks	EH VitD: 25(OH)D=21.8 ± 5.4ng/ml EH VitD: PTH =53.5 pg/ml EH Placebo: 25(OH)D=20.4 ± 5.7ng/ml EH Placebo: PTH = 53.8 pg/ml	A supplement of vitamin D led to a significant reduction of plasmatic aldosterone, but no effect on renin or aldosterone to renin ratio
Ismail NA (2020) (51)	PA: 9♀ - 8♂ (42 ± 8 yrs) 2400 IU/day vitamin D supplementation for 3 months	APA: 25(OH)D=21.8 ± 12.4 ng/ml APA: iPTH =4.74 ± 4.43 pmol/L 70% of PA patients has significant vitamin D insufficiency. 3 months of vitamin D treatment resulted in a significant improvement in the 25(OH)vitamin D level	Vitamin D treatment leads to a significant reduction in the plasma aldosterone concentration, systolic blood pressure, as well as improvement in the eGFR. Vitamin D treatment improves all of these interrelated parameters possibly suggesting an interplay between vitamin D, aldosterone and kidney function

VDR: vitamin D receptor; 1,25(OH)D: 1,25-dihydroxyvitamin D; 1,25(OH)2D3: 1α,25-dihydroxyvitaminD3; CRE: cAMP response element; CREB, CRE-binding protein; CBP: CREB-binding protein; ♀: female; ♂: male; RAS: renin angiotensin system; PA: primary aldosteronism; EH: Essential Hypertension; 25(OH)D: 25-hydroxyvitamin D; PTH: parathyroid hormone; MR: mineralocorticoid receptor; APA: aldosterone-producing adenoma; nPA: without primary aldosteronism; BMD: bone mineral density; NFA: nonfunctioning adrenal adenoma; iPTH: intact parathyroid hormone; eGFR: estimated glomerular filtration rate.

The study by Li et al. shows that vitamin D works as a negative endocrine regulator of the renin-angiotensin system in animals. In the mice, in fact, with null vitamin D receptor (VDR-null) the expression of renin and angiotensin II has increased and it leads to hypertension, cardiac hypertrophy, and an increase in the intake of water. In wild-type mice, the inhibition of the synthesis of 1,25(OH)2D leads to an increase in the renin expression, while the injection of 1,25(OH)2D leads to renin suppression. Thus 1,25(OH)2D dramatically suppresses renin transcription thanks to a mechanism mediated by VDR in cellular cultivation (39). Other studies on animals show that VDR, linked to its ligand 1,25(OH)2D, negatively regulates the renin (39, 40).

Cross -sectional studies confirmed a connection between vitamin D metabolites and renin-angiotensin-aldosterone system (RAAS): 25(OH)D levels are inversely linked to plasma renin activity (PRA) as high circulating levels of angiotensin II (41). All these confirm that there’s more RAAS activation in case of vitamin D deficiency (52).

A study by Bi C et al. aimed at understanding endocrine mechanisms involved in APA formation, through the analysis of biological processes and signaling pathways related to APA. In this study 19 endocrine genes have been found that are involved in regulatory pathways but only VDR, POR, and RET were differentially expressed in APA specimens. Thus VDR would represent the most relevant transcription factor, and its target genes including CYP11B2 and KCNJ5 would play an important role in the endocrine mechanism of APA (46).

Moreover, a study by Lundqvist et al. showed as 1α,25-OHD2 plays a role in hormone production and in the expression of the steroidogenic enzymes that are crucial in the human adrenocortical cell line NCI-H295R; it has shown that cell treatment with 1α,25-OHD2 suppressed levels of corticosterone, aldosterone, DHEA, DHEA-sulphate and androstenedione in the culture medium (42).

Apart from molecular and *in vitro* studies, we analyzed clinical studies aimed at searching for a correlation between vitamin D and PA. The results of these studies are not unique. In fact, several studies reported that vitamin D levels were not different between patients with PA and essential hypertension (EH) (43, 44).

Moreover, Ceccoli et al. analyzed the picture of secondary hyperaldosteronism to the increase in the urinary excretion of calcium by comparing 116 patients with PA and 110 patients with EH. After medical or surgical treatment in 40 patients with PA, bone mineral density (BMD) significantly increased in the lumbar spine, femoral neck, and total hip; PTH levels were significantly reduced, and instead, 25OH vitamin D levels were unchanged before and after the treatment (25-OH vitamin D before treatment: 24 ± 16 ng/ml after treatment: 26 ± 12 ng/ml p value n.s.). In addition, there wasn’t a statistically significant difference between 25-OH vitamin D levels in both groups (PA ed EH) (47).

Another study by Salcuni et al. considered 188 people with adrenal incidentaloma, who were observed between November 2009 and October 2011. After confirmatory testing: 11 patients were diagnosed with PA and 15 patients were later taken as controls (nPA). Vitamin D levels were deficient in both groups, in PA group they were lower but not statistically significant. PA was associated with low bone mass, increased prevalence of osteoporosis, and vertebral fractures. So aldosterone excess lead not only to cardiovascular and renal injury but also to bone damage (45). Similarly, the study by Zhang et al. that recruited 142 patients with adrenal adenoma (84 with APA and 58 with non-functional adrenal adenoma (NFA) showed that vitamin D levels were low in both the two groups, and particularly in APA group the levels were slightly lower. Instead, the serum PTH levels were significantly higher in APA, thus PTH may be a promising auxiliary index for the clinical diagnosis of APA (49).

As we can see, the results are not clear and we should not forget the possibility, supported by many works, according to which vitamin D itself can play a role in EH pathogenesis: a great number of observational studies support a datum that vitamin D could have a protective effect against hypertension development (53).

A Mendelian randomization study of Vimaleswaran et al. has shown, in fact, a correlation between 25(OH)vitamin D concentration, blood pressure, and hypertension risk. The results of this study suggest that people who have genetic variation linked to low endogenous production of 25(OH)vitamin D have an increased risk of hypertension, it underlines the need for more randomized, controlled, and well-planned studies to assess the potential clinical benefits of vitamin D reintegration (54).

In literature, we have also found different authoritative studies that have found a link between vitamin D deficiency and hyperaldosteronism. For example, a randomized controlled study by Grübler MR performed on 188 patients with arterial hypertension and poor vitamin D levels, has shown that a supplement of this vitamin led to a significant reduction of plasmatic aldosterone compared to a control group with placebo (50). Other studies on patients with heart failure, to whom Vitamin D was administered, have shown a reduction in plasmatic aldosterone levels (55, 56). Moreover, the aim of the study by Petramala et al. was to evaluate the impact of an excess of aldosterone on mineral metabolism and BMD. Patients with PA had a higher plasmatic PTH, lower 25(OH)vitamin D serum levels, a higher prevalence of vitamin D deficiency, and a higher prevalence of osteopenia/osteoporosis compared to EH and controls (48).

A recent study by Ismail et al. (51) concluded that 70% of the patients with PA showed a significant deficiency in vitamin D. After a 3 months treatment with vitamin D determined: 1) a significant improvement of 25(OH) vitamin; 2) a significant decrease of aldosterone plasmatic concentration; 3) decrease of systolic blood pressure; 4) improvement of eGFR. Vitamin D treatment improved all these parameters, thus probably suggesting an interaction between vitamin D, aldosterone, and kidney function.

Ismail’s study certainly had some limits (for example the small sample size; observational, uncontrolled, nonblinded design; lack of a randomized design ensuring a balanced distribution of confounders between patients receiving vitamin D or a placebo), nevertheless, the obtained results are very interesting and promising, and they should be a starting point for new studies to investigate such a fascinating correlation between vitamin D, hyperaldosteronism and blood hypertension (57).

## Vitamin D and primary adrenal insufficiency

Primary Adrenal Insufficiency (PAI) is a rare but severe-threatening disease in children and adults; the diagnosis is frequently delayed. PAI is defined by the inability of the adrenal cortex to produce sufficient amounts of glucocorticoids, mineralocorticoids and adrenal androgen hormones. This condition was first described by Thomas Addison and for this reason it is also known as Addison’s disease (AD). It is a potentially critical condition due to the central role of these hormones in energy and fluid homeostasis. Cortisol deficiency results in a decrease in feedback to the hypothalamic-pituitary axis and subsequent enhanced stimulation of the adrenal cortex by elevated levels of plasma ACTH. Consequent to disruption of adrenal mineralocorticoid synthesis, renin release by the juxtaglomerular cells of the kidneys increases. The signs and symptoms of PAI are mainly based on the deficiency of gluco- and mineralocorticoids and they are: weakness, fatigue, hyponatremia, hyperkalemia, depression, anxiety, weight loss, abdominal pain, musculoskeletal pain, orthostatic hypotension, changes in blood count (anemia, eosinophilia, lymphocytosis), hypoglycemia, hyperpigmentation of the skin, loss of axillary and pubic hair (58). In adulthood, the most common etiologies include: autoimmune, hemorrhage, infiltrative disorders, metastases and infection, whereas congenital adrenal hyperplasia (usually due to 21‐hydroxylase deficiency) affects approximately 1:18000 infants and children (59). Although the pathogenesis of AD has not been made completely clear, a role of interaction between HLA aplotypes and environmental factors has been postulated: environmental factors seem to act as triggers in a context of genetic susceptibility (HLA), all of this leads to the destructive infiltration of the adrenal cortex by CD8-T lymphocytes and to the typical production of 21-OHase antibodies (60). The correlation between AD and vitamin D is really fascinating and can even be found at a genetic level: various genes responsible for metabolizing vitamin D and polymorphisms of the VDR gene are involved in the onset of AD (61, 62).

The most important studies evaluating the Vitamin D and Primary Adrenal Insufficiency are reported in **Table 4**.

**Table 4:** Main characteristics of the 10 studies included in the review on Primary Adrenal Insufficiency and Vitamin D.

Study	Study Characteristics	Main results	Conclusions
Pani MA (2002) (61)	Addison’s disease: 71♀ - 24♂ Controls: 139♀ - 81♂ DNA was isolated from whole blood and were genotyped for VDR polymorphismsFok I, BsmI, Apa I and Taq I	The genotypes ‘ff’ and ‘tt’ appear to be associated with susceptibility to Addison’s disease	The results conclude that the VDR genotype is linked to Addison’s disease
Ramos Lopez E (2004) (63)	Addison’s disease = 124 analyzes two polymorphisms with a single nucleotide in the hydroxylasis gene CYP27B1 for a link with Addison’s disease	The polymorphism C/A of the promotor CYP27B1 (21260) seems to be linked to endocrinal autoimmune diseases.	These results suggest a regulatory difference of the CYP27B1 hydroxylasis to predispose endocrinal autoimmunity
Jennings CE (2005)(64)	AAD: n=104 Controls: n=464 examined three SNPs that are located in the 58 region and promoter of the CYP27B1 gene for association	All three of the CYP27B1 SNPs were associated with AAD The C allele at the -1260 promoter SNP was the allele most associated with AAD	CYP27B1 promoter allele is associated with autoimmune Addison’s disease, and extend this finding to include an associated promoter haplotype.
Fichna M (2010) (62)	Addison’s disease: 72♀ - 29♂ Controls: 173♀ - 78♂ to investigate the associations of CYP27B1 C(−1260)A and PDCD1 G7146A polymorphisms with Addison disease	The CYP27B1 C(− 1260) allele appeared significantly more frequent in AAD compared to controls	This study confirms the association of the CYP27B1 C(−1260)A polymorphism with Addison disease, whereas the contribution of PDCD1 G7146A seems less likely
Ramagopalan SV (2013) (65)	Addison’s disease N= 12	Vitamin D deficiency	In patients with hypovitaminosis D there are significantly higher levels of Addison’s disease and other autoimmune diseases
Korwutthikulrangsri M (2015) (66)	Patients n = 32 Controls n = 36 studies the interrelationship between vitamin D deficiency and and its relationship with adrenal function children in critical conditions	patients: 25(OH)D=16.6 ng/ml controls: 25(OH)D=24.2 ng/ml	50% of the children in critical conditions on ICU showed a total concentration of 25(OH)D in the blood inferior to 50 nmol/L. Vit D deficiency was correlated to increased mortality. No link is found between the 25(OH)D serum and suprarenal function
Pazderska A (2016) (67)	UK ADD: 312♀ - 103♂ (38 yrs) Polish ADD: 167♀ - 64♂ (36 yrs)	month of birth exerts an effect on the risk of developing AAD	Hypovitaminosis D, together with the exposition to seasonal viral infections, could have deregulated innate immunity, increasing the risk of the insurgence of AD
McNally J (2013) (68)	Pediatric ICU children: n= 319	25(OH)D= 40.3 nmol/L no association was found between vitamin D status and AI.	No link whatsoever was found between the state of the vitamin D and IA. Vitamin D deficiency would contribute to exacerbating the effect of IA on cardiovascular stability in children in critical conditions
Penna-Martinez M (2018) (69) Cross-over study	AD: 7♀ - 6♂ (48 yrs) AB arm: 4000UI cholecalciferol for 3 months + placebo for 3 months BA arm: placebo for 3 months + 4000UI cholecalciferol for 3 months	25(OH)D basale = 15.8 ng/ml 25(OH)D after cholecalciferol=41.5 ng/ml late-activated Th and late-activated Tc cells decreased; monocytes increased after VD therapy. T-cell changes were associated with 2 polymorphisms (CYP27B1-rs108770012 and VDR-rs10735810), but no changes in the 21-hydroxylase antibody titers were observed	This pilot study provides new information about the immunomodulation of vitamin D in AD. Three months of treatment with cholecalciferol achieved sufficient 25(OH)D3 levels and can regulate late-activated T-cells and monocytes in patients with AD
Zawadzka (2021) (70)	AD: 25♀ - 6 ♂ (44.2 ± 14.6 yrs)	AD: 25(OH)D= 18.1 ± 9.95 ng/mL AD: PTH =38.6 ± 20.7 pg/mL The mean serum VD concentration was significantly lower in patients with severe fatigue and limited exercise capacity.	This study demonstrates a high incidence of VD and a significant correlation between low levels of VD and severe fatigue in AD

♀: female; ♂: male; VDR: vitamin D receptor; AAD: autoimmune Addison’s disease; SNOs: single nucleotide polymorphisms; 1,25(OH)D: 1,25-dihydroxyvitamin D; 1,25(OH)2D3: 1α,25-dihydroxyvitaminD3; 25(OH)D: 25-hydroxyvitamin D; ICU: intensive care unit; UK: United Kingdom; AD: Addison’s disease; IA: adrenal insufficiency; PTH: parathyroid hormone; VD: vitamin D deficiency.

The study of Pani et al. starts from the assumption that variants of the VDR were associated with type 1 diabetes and thyroidal autoimmunity and goes on to analyze the VDR polymorphisms in Addison’s disease. 95 patients and 220 controls were genotyped for VDR polymorphisms (Fok I, BsmI, Apa I e Taq I). The results demonstrated that the genotypes ‘ff’ e ‘tt’ were significantly more frequent in patients with PAI compared to the controls. The distribution of the genotype BsmI also differed significantly between the patients and the controls. It was therefore concluded that the VDR genotype was linked to Addison’s disease (61).

The study by Lopez et al. analyzed two polymorphisms with a single nucleotide in the hydroxylasis gene CYP27B1 for a link with AD, Hashimoto’s thyroiditis, Graves’ disease and type 1 mellitus diabetes. CYP27B1 hydroxylasis catalyzes the conversion of the 25OHD3 in 1,25(OH)2D3, that plays a role in immunity regulation and cell proliferation. One hundred twenty-four patients with AD were taken into consideration. A significant link has been found between allelic variation of the promotor (21260) C/apolimorphism and AD, concluding therefore that the polymorphism C/A of the promotor CYP27B1 (21260) seems to be linked to endocrinal autoimmune diseases. These results suggest a regulatory difference of the CYP27B1 hydroxylasis to predispose endocrinal autoimmunity (63). Moreover, this link was also later confirmed by the study by Ficna M et al. in 2010 (62)

Successively, Ramagopalan et al. studied the potential role of hypovitaminosis D in influencing the pathogenesis of immuno-mediated diseases (65). The authors observed that in the patients with hypovitaminosis D there were significantly higher levels of AD and other autoimmune diseases.

Korwutthikulrangsri et al. (66), studying the interrelationship between vitamin D deficiency and PAI in children in critical conditions, observed that about 50% of the children on entering intensive care showed a total concentration of 25(OH)D in the blood inferior to 50 nmol/L. Furthermore this condition of deficiency was correlated to the gravity of the illness, multiorgan dysfunction and increased mortality. Instead, no link was found between the 25(OH)D serum and adrenal function. Moreover, a study by McNally et al (68), conducted on 319 children in six Canadian pediatric intensive care units evaluated the potential relationship between vitamin D levels, the adrenal state and cardiovascular dysfunction linked to PAI. No link whatsoever was found between the state of the vitamin D and PAI. However, the cardiovascular dysfunction linked to PAI seemed to be influenced by the state of the vitamin D; in fact, the PAI in patients with vitamin D deficiency was linked to significantly higher risk of cardio vascular dysfunction. Another study by Pazderska et al. later demonstrated that people born in winter ran a greater risk of suffering from AD. These results suggested therefore that hypovitaminosis D, together with the exposition to seasonal viral infections, could have deregulated their innate immunity, increasing the risk of the insurgence of AD (67).

The study by Martinez M et al. represents the first randomized crossover study which investigates the integration of cholecalciferol in patients with AD. The work included 13 patients with AD who received reintegration with cholecalciferol (4000 UI/day) for 3 months. The following were monitored: the plasmatic levels of 25-OHD3 and the immunitary cells, and monocytes. The exploratory analysis included the correlation of the changes with the genic polymorphs correlated to the vitamin D and the antibody titles of the 21- hydroxylasis (69). The median concentrations of 25(OH)D3 were significantly increased after 3 months of treatment with cholecalciferol. Within the T cells, only the tardily activated T cells and the Tc cells activated tardily had diminished, while the monocytes had increased after the therapy with vitamin D. The changes in the T lymphocytes were linked to two polymorphisms (CYP27B1-rs108770012 and VDR-rs10735810); no changes were observed in the antibody titles of the 21-hydroxylasis. It was concluded that in 3 months of treatment with cholecalciferol the patients: 1. reached sufficient levels of 25(OH)D3; 2. this reintegration led to the regulation of the tardy activation of the T lymphocytes and of the monocytes in patients with AD. The esplorative analysis revealed potential genetic contributions. This pilot study provides new information about the immunomodulation of vitamin D in AD.

Another recent study by Zawadzka et al. (70) retrospectively analyzed medical records of 31 adult patients with the diagnosis of autoimmune AD and demonstrate a high incidence of vitamin D deficiency and a significant correlation between low levels of vitamin D and severe fatigue as well as limited exercise capacity in AD patients.

It can therefore be concluded that the data from the studies carried out up to now would suggest that hypovitaminosis D and AD could be correlated; this correlation would also be endorsed by the potential role of vitamin D in modulating the immune response and by the autoimmune nature of AD. New longitudinal clinical studies could further confirm this data and maybe give us further news on the question of a possible use of vitamin D in the prevention or as an adjuvant therapy in this illness as in other immune-based pathologies.

## Conclusions

To sum up the studies carried out so far on the link between vitamin D status and adrenal gland diseases, although relatively scarce and heterogeneous, allow us to formulate the following considerations.

- In patients with Cushing’s disease, levels of 25OHD are lower than in control subjects and appear to be negatively correlated with urinary cortisol levels.
- Reduced levels of vitamin D and under-expression of VDR seem to have a role in tumors of the adrenal cortex.
- Several studies have confirmed that a vitamin D deficiency is associated with an activation of the RAAS system; furthermore, some data from the literature have suggested that the administration of vitamin D may modulate hyperaldosteronism and the consequent hypertension.
- Vitamin D due to its ability to modulate the immune response could play a protective role in the pathogenesis of an autoimmune disease such as Addison’s disease

Today the view that hormones have only one function is outdated. Thanks to thousands of molecular, biochemical and clinical studies, it has been observed that there is a correlation between hormones and cardio-vascular risk, autoimmunity, inflammation.

Vitamin D has been a surprising discovery in recent years and its many functions are even more surprising. The studies analyzed in this work are promising: they support a complex network of interactions between adrenal hormones and vitamin D, with implications in prevention and health. So after this long journey through clinical studies, hormones and biochemistry, let’s sum it up and go back to our original question: adrenal gland and vitamin D, myth or reality?

We have evidence that this link is more than just a myth. The topic is vast and fascinating, the potential is great: there would be the possibility of using this vitamin for therapeutic or even preventive purposes in adrenal diseases. Evidences seem to support a role of vitamin D deficiency in the adrenal diseases onset but nowadays they are limited and not conclusive, thus not allowing to formulate diagnostic and therapeutic conclusions for each of these pathologies.

Evidence on potential efficacy of vitamin D administration in clinical practice for patients with adrenal diseases is currently lacking, we have not confirmed results about quantity and duration of vitamin D supplementation or how vitamin D could change the development, the course and the prognosis of the adrenal pathologies examined.

It would be very interesting to deepen these clinical implications with new future studies and works.

## Author contributions

AA and CC conceived the idea, AA, LB and MD drafted the manuscript, SG and CC supervised the process and contributed to editing. All authors contributed to the article and approved the submitted version.

## Conflict of interest

The authors declare that the research was conducted in the absence of any commercial or financial relationships that could be construed as a potential conflict of interest.

## Publisher’s note

All claims expressed in this article are solely those of the authors and do not necessarily represent those of their affiliated organizations, or those of the publisher, the editors and the reviewers. Any product that may be evaluated in this article, or claim that may be made by its manufacturer, is not guaranteed or endorsed by the publisher.
